# Missed Until Critical: Unravelling the Mystery of Persistent Hemolysis With a Definitive Diagnosis of Babesiosis

**DOI:** 10.7759/cureus.86381

**Published:** 2025-06-19

**Authors:** Paris D Slagle, Shirley Ann T Tomdio, Lauren A Waldorf, Eric A Williams, Habeeb Agbabiaka, Gashaw Hassen, Vaughn E Powell, Jada Harris

**Affiliations:** 1 Internal Medicine, University of Maryland Capital Region Medical Center, Largo, USA; 2 Progressive Care, Mercy Medical Center, Baltimore, USA; 3 Medicine and Surgery, Parma University, Parma, ITA; 4 Medicine, Addis Ababa University, Addis Ababa, ETH

**Keywords:** babesiosis, complication, hemolysis, ixodes scapularis tick, malaria, maltese cross, misdiagnosis

## Abstract

Babesiosis is a parasitic protozoan infection caused primarily by *Babesia microti* in the United States. Its presentation can imitate that of malaria, which often leads to misdiagnosis. We present here a case of a 54-year-old female with multiple comorbidities, such as hypertension and hyperlipidemia, who was initially diagnosed with malaria but, with further investigation, was found to have babesiosis. The patient initially presented with anemia and thrombocytopenia, prompting an extensive and meticulous workup including a peripheral blood smear and autoimmune studies. From these tests, we were able to diagnose her with a malarial infection that was treated with quinine and doxycycline, then atovaquone/proguanil following worsening infection. Despite the appropriate treatment, she developed septic shock and respiratory failure, prompting a higher level of care in the intensive care unit. With persistent parasitemia, a sample was sent to the department of health for speciation, which determined that she was infected with Babesia. Clinically, she improved after the transition to atovaquone and azithromycin, but unfortunately, she was readmitted due to persistent hemolysis and pneumonia requiring extended antibiotic therapy and supportive care. This case highlights the challenges posed by the similarities of babesia and malaria infection and emphasizes the importance of geographical location and confirmatory testing in differentiating these two infections. It raises awareness among clinicians regarding tick-borne illnesses in endemic regions, as in this case, to minimize delays in diagnosis and treatment. This case also shows how early recognition and treatment are crucial to prevent severe complications of babesiosis, including septic shock, especially in the immunocompromised and elderly population.

## Introduction

Babesiosis is a tick-borne protozoal infection caused by Babesia species, primarily *Babesia microti*, in the United States. It is transmitted through the bite of Ixodes scapularis, the same vector responsible for Lyme disease [1]. It mainly affects red blood cells (RBCs), leading to hemolysis and a malaria-like febrile illness. While often asymptomatic in healthy individuals, babesiosis can cause severe complications when treatment is delayed and in asplenic, immunocompromised, and persons over the age of 50.

Babesiosis shares striking similarities with malaria, as both are caused by intraerythrocytic protozoa (Babesia spp. and Plasmodium spp., respectively). These parasitic diseases present with overlapping clinical symptoms, including fever, chills, fatigue, and hemolytic anemia [2]. Microscopically, both infections reveal intraerythrocytic parasites on peripheral blood smears (PBS), presenting further opportunity for potential diagnostic pitfalls. However, unlike malaria, which is transmitted by Anopheles mosquitoes and is more common in tropical regions [2], babesiosis is endemic to certain temperate areas, particularly the northeastern and upper Midwestern United States. The treatment of babesiosis also differs from malaria, as it relies on a combination of atovaquone and azithromycin or, in severe cases, clindamycin and quinine [3].

This case report follows a patient in the northeastern United States who presented with signs and symptoms of anemia and was ultimately diagnosed with babesiosis. However, her condition was initially mistaken for malaria. The case stresses the diagnostic challenges posed by the similarities between these two infections. It emphasizes the importance of considering geographic exposure, travel history, and confirmatory testing to ensure accurate diagnosis and appropriate management. Furthermore, severe babesiosis can lead to long-term complications, including persistent hemolytic anemia, post-infectious fatigue, splenic infarction, and in rare cases, chronic parasitemia in immunocompromised individuals. Recognizing these potential sequelae is critical for monitoring patients beyond the acute phase of the illness and preventing further complications. This case report will highlight a diagnostic challenge and the complications that can occur thereafter.

## Case presentation

First admission

On the first admission (09/23/24-10/01/24), the patient presented as a 54-year-old female with a significant past medical history of diabetes mellitus (DM), hypertension (HTN), chronic kidney disease (CKD) stage IV, hyperlipidemia, and diabetic neuropathy. She arrived at the emergency department (ED) at the University of Maryland Capital Region Medical Center (UMCAP) on (09/23/2024) with chest pain and occasional shortness of breath. Additionally, she experienced numbness and tingling that began a week prior. She also presented with dizziness that led to balance issues, causing her to fall and fracture her wrist. Upon admission, laboratory tests, shown in Table 1 below, revealed that the patient was anemic, with a hemoglobin (Hgb) level of 8.3 (normal range for females >12). Evaluation for possible acute coronary syndrome was negative. Based on her Hgb, anemia was found to be the inciting factor, prompting further investigation. The anemia workup included a complete blood count and iron studies, which showed a ferritin level of 260, total iron binding capacity of 25, RBC of 3.06, Hgb of 7.8, hematocrit (Hct) of 25.9, and platelet (PLT) count of 112. Over the subsequent days, the patient's Hgb continued to decline, reaching 6.5, with a platelet (PLT) of 104. At this point, it was decided to transfuse the patient with one unit of packed red blood cells (PRBC) while investigating potential autoimmune causes for her anemia and thrombocytopenia. An antinuclear antibody (ANA) test and GAD-65 antibody were ordered, and a gastrointestinal (GI) consult was requested to assess for possible GI bleeding. Post-transfusion, the patient’s Hgb increased to 7.7, then 8.5, and remained stable. The ANA immunoglobulin (Ig)G by ELISA returned abnormal results, showing a cytoplasmic pattern with titers of 1:640, consistent with anti-mitochondrial (AMA) antibodies, suggesting primary biliary cholangitis (PBC) or systemic sclerosis (SSC). The GAD-65 antibody measured <5 (reference range 0.0-5.0 IU/mL). 

**Table 1:** Pertinent laboratory values from first admission Hgb=hemoglobin, Hct=hematocrit, RBC=red blood cell, PLT=platelets, LDH = lactate dehydrogenase, TIBC=total iron binding capacity, GAD-65=glutamic acid decarboxylase 65, ANA=Antinuclear Antibody, ADAMTS13=A Disintegrin And Metalloproteinase with Thrombospondin type 1 motif, member 13, (-)=negative, (+)=positive

Lab/Test	Values					Reference range
	Day 1 (admitted)	Day 2-3	Day 4-5	Day 6	Day 7 (discharged)	
Hgb	8.3	7.8 -> 6.5	7.7 -> 8.5 (post-transfusion)	5.8 -> 8.7 -> 11.2 (post-transfusion)	9.9 -> 8.7 -> 9.0	12-14 g/dL
Hct	—	25.9	—	—	—	41-47%
PLT	—	112 -> 104	—	—	—	150-475 10*3/uL
RBC	—	3.06	—	—	—	3.8-5.10
Reticulocyte	—	—	—	3.0%	—	0.6-1.5%
Haptoglobin	—	—	—	<14	—	30-200 mg/dL
LDH	—	—	—	635	—	135-214 unit/L
Ferritin	—	260	—	—	—	24-307 ng/mL
TIBC	—	25	—	—	—	240-450 mcg/dL
ADAMTS13	—	—	—	80%	—	70-100%
GAD-65	—	—	<5	—	—	0.0-5.0 IU/mL
ANA	—	—	1:640	—	—	0 or 1:20 (-), 1:40 (weakly +), 1:160 to 1:320 (Moderately +), 1:640 or higher (Strongly +)

The remainder of her hospital stay was insignificant, and her Hgb remained stable, though it gradually decreased from 11.2 to 9.9, then to 8.7, and stabilized to 9.0 just before discharge. It is important to note that a peripheral blood smear was done the day before discharge, given that the patient's Hgb continued to gradually trend down. At this point, ADAMTS13 (a disintegrin and metalloproteinase with thrombospondin type 1 motif, 13) for thrombotic thrombocytopenic purpura (TTP) had not been identified, and we wanted to further investigate the patient's down-trending Hgb. Hematology was consulted and recommended that the patient follow up with their department and primary care physician (PCP) for ongoing central blood cell count (CBC) monitoring and multiple myeloma workup. The patient was then discharged after spending a total of seven days in the hospital with the diagnosis of uncontrolled DM and severe symptomatic hemolytic anemia.

Between admissions

After being discharged on the first of October, the patient visited the University of Maryland Laurel Medical Center Emergency Department (UMLMCED) the following day due to complaints of nausea and concerns for low blood sugar. She reported experiencing runny stools for the past three days and chills the day before. She denied having a fever, chest pain, vomiting, dysuria, or lightheadedness. During the physical examination, severe tenderness was noted in the right upper quadrant and epigastric area. Laboratory tests, shown in Table 2 below, revealed a Hgb level of 9.5 and a PLT of 66. The automated differential of PBS showed the presence of atypical lymphocytes, schistocytes, abnormal RBC morphology, abnormal PLT morphology, and giant PLT. An abdominal ultrasound indicated hepatomegaly with multiple non-shadowing stones or sludge balls and a positive Murphy sign. The patient received a normal saline bolus and was sent home on 10/04/24 with Zofran for nausea. She was advised to return to the emergency department if her pain worsened despite treatment. 

**Table 2:** Pertinent laboratory values between admissions Hgb=hemoglobin, PLT=platelets

Lab/Test	Values		Reference range
	Day 1 (admitted)	Day 4 (discharge)	
Hgb	9.5	8.5	12-14 g/dL
Plt	66	80	150-475 10*3/uL

Later that night, physicians at UMLMCED received a call from their lab reporting that a diagnostic malarial smear was done due to high clinical suspicion for malaria and abnormal CBC, and that the patient should be transferred to UMCAP for malaria testing. The attending physician left voicemails after several unsuccessful attempts to reach her, as she had been discharged before these findings. 

Second admission

On the second admission (10/05/24-10/31/24), the patient returned to the ED at UMCAP one day after she was seen at UMLMCED with complaints of chronic left lower back pain, right upper quadrant pain, dizziness, a headache accompanied by poor oral intake for a couple of days. The surgical team evaluated her for acute cholecystitis and recommended starting Zosyn® while hospitalized, with a follow-up for an elective cholecystectomy. She was informed of the positive malaria result, and reported that she had not travelled outside the state of Maryland. At this time, the infectious disease (ID) team was consulted. The ID team recommended the patient be started on quinine sulfate, taking two tablets three times daily in addition to doxycycline 100 mg intravenous (IV) twice daily for one week, with a repeat smear scheduled for two days later. 

Laboratory results and vital signs remained stable until the third day of hospitalization. On day three of this admission, the rapid response team was called for hypotension(systolic blood pressure in the 80s), bradycardia (junctional rhythm on ECG), and hypoxia (oxygen saturation of 93%). Given her rapid decline, she was then admitted to the ICU on an epinephrine (levophed) drip, intubated, and both quinine and doxycycline were held for the day. Per ID team recommendation, artemether/lumefantrine (Coartem®) was initiated the following day in place of doxycycline and quinine, as these were thought to be a cause of hypotension. Over the subsequent days, the patient remained intubated, requiring pressure support with Levophed®, and was diagnosed with septic shock secondary to severe malaria infection. On the fifth day, the malarial smear test with microscopy continued to test positive, and the ID team recommended restarting doxycycline 100 mg twice daily. Unfortunately, no polymerase chain reaction (PCR) or rapid tests were done. The following day, the patient became unresponsive to vocal and painful stimuli, leading to the decision to hold sedation for further evaluation. A chest X-ray at this time indicated a possible superimposed infection or increased pulmonary vascular congestion. She was administered furosemide 20 mg and showed mild improvement.

On day seven, the patient was extubated but experienced tachypnea and increased work of breathing, resulting in her being placed on bilevel positive airway pressure (BiPAP). By day eight, malarial smears were still positive, and ID recommended that the patient be placed on atovaquone/proguanil (Malarone®) once daily for three days. However, her condition worsened, leading to respiratory distress with cardiogenic shock, requiring reintubation. Imaging revealed the development of hospital-acquired pneumonia, prompting the initiation of additional antibiotics for management. On day eleven, the patient’s smear still tested positive for malaria, and a sample was sent to the health department for speciation. At this point, the parasite was identified as Babesia rather than malaria, indicating that the patient was initially misdiagnosed. As a result, doxycycline and atovaquone/proguanil were discontinued, and she was started on atovaquone and azithromycin for seven days. 

The patient remained stable until day seventeen, when her Hgb dropped to 6.9, as seen in Table 3 below. She received a transfusion of one unit of PRBC, elevating her Hgb to 9.3. Her antibiotic regimen for Babesia was completed by day 18. On day 20, she was extubated and transferred to the medical-surgical unit. The patient remained hemodynamically stable for the remainder of her hospital stay, and she was discharged home 11 days later on supplemental oxygen with instructions to follow up with her primary care physician. It is important to note that the patient remained afebrile during her entire hospital stay, as supported by the records. She had a Glasgow coma scale (GCS) score of 15 on initial arrival and was sedated and intubated for respiratory failure, but no documented serial GCS score could be obtained during chart review. Liver function tests (LFTs) were normal despite AMA and ANA being positive. The patient also has a past medical history of CKD stage IV, and her initial labs revealed creatinine (Cr) 2.0, blood urea nitrogen (BUN) 99, and glomerular filtration rate (GFR) of 29, as indicated in Table 3 below. In deep review, this was her baseline kidney function.

**Table 3:** Pertinent laboratory values from the second admission Hgb=hemoglobin, PLT=platelets, BUN=blood urea nitrogen, Cr=creatinine, GFR=glomerular filtration rate

Lab/Test	Values			Reference range
	Day 1 (admitted)	Day 17	Day 26 (discharge)	
Hgb	8.5	6.9 -> 9.3 (s/p transfusion)	7.6	12-14 g/dL
Plt	80	98	355	150-475 10*3/uL
BUN	99	65	60	6-23 mg/dL
Cr	2.0	2.3	2.0	07-1.2 mg/dL
GFR	29	25	29	>60 mL/min

Third admission

After the previous admission, the patient remained home for approximately 30 days and was following up with her PCP. Approximately two months (11/30/24-12/12/24) after the first hospitalization, she presented to the emergency room (ER) with worsening cough, shortness of breath, and chest pain requiring high flow oxygen on nasal cannula (HFNC). Chest X-ray obtained the following day showed underlying pneumonia with vascular congestion, suggesting pulmonary edema. Given her recent history of babesia, the decision was made to restart atovaquone, azithromycin, and add clindamycin for pneumonia secondary to recurrent babesiosis. ID was consulted and recommended that she remain on her current antibiotic regimen, but add cefepime for complete coverage for hospital-acquired pneumonia. Venous blood gases (VBG) at that time showed she was becoming acidotic. Two days later, her anion gap was beginning to close, though her VBG was still positive for acidosis. The following morning, she had increasing HFNC requirements and refused a ventilation/perfusion scan (V/Q scan) recommended by the treatment team to assess for pulmonary embolism. It was then decided to diurese 3L of fluid via furosemide, given imaging was positive for possible pulmonary edema. She eventually agreed to perform a V/Q scan, which was negative. She was soon able to begin the weaning process off HFNC. One week into this hospital stay, she was officially off HFNC, tolerating a nasal cannula well, and was walking around the unit. At this point, she was still being diuresed as this was a regimen added that improved the patient’s overall status. She was then downgraded from the ICU to the floor, where she remained stable. She completed her antibiotic course and was discharged.

Differential diagnosis

In diagnosing the patient, we began by considering the most common cause of anemia in individuals over 50 years of age: gastrointestinal (GI) bleeding, as seen in Table 4. Therefore, we referred the patient to the GI department as part of our anemia workup. However, the investigation ruled out GI bleeding as a source of her anemia, despite her having reported black, tarry stools days before her visit to UMLMCED. 

**Table 4:** List of differential diagnoses with identifying features TTP=Thrombotic Thrombocytopenic Purpura,  LDH=Lactate dehydrogenase, WBC=White blood cell, AMA=Anti-mitochondrial antibody, ANA=Antinuclear Antibody, GI=Gastrointestinal, ADAMTS13=A Disintegrin And Metalloproteinase with Thrombospondin type 1 motif, member 13, N/A=Not Applicable, (-)=negative, (+)=positive

Differential	Clinical symptoms	Labs		
		CBC	PBS	Special Testing
GI Bleed	Black tarry stool, Symptoms of anemia (pallor, tachycardia, tachypnea, hypotension)	Severe anemia with values as low 5.8	N/A	Colonoscopy (-) for bleed
TTP	Petechiae and bruising	Thrombocytopenia	Schistocytes on PBS	ADAMTS13 (-)
Autoimmune hemolytic anemia	Symptoms of anemia (pallor, tachycardia, tachypnea, hypotension)	High LDH Low haptoglobin	N/A	Direct Coombs (-) ANA (+) AMA (+)
Infectious (Babesia)	Fever, leukocytosis, anemia	Elevated WBC High LDH Low haptoglobin	Intra-erythrocytic rings “Maltese cross”	Blood cultures negative
Infectious (Malaria)	Fever, leukocytosis, anemia	Elevated WBC High LDH Low haptoglobin	Intra-erythrocytic rings	Blood cultures negative

As her anemia worsened, we investigated potential autoimmune causes due to thrombocytopenia. Both ANA and AMA antibodies tested positive, which can account for the patient’s anemia. However, the diseases associated with these titers typically manifest with other features, including, but not limited to, elevated liver enzymes and renal abnormalities. Evidence of gallbladder stones and sludge possibly indicates that there was some sort of liver pathology, associated with AMA (ex., primary biliary cholangitis), that had occurred. The presence of these immune titers prompts investigation for future diagnosis, though they were not clinically relevant to the patient’s presenting illness. Ultimately, the PBS showing intraerythrocytic rings suggestive of babesia, though initially it was thought to be malaria, led to the diagnosis. Furthermore, malaria and babesia both present with signs of anemia, high LDH, and low haptoglobin as RBCs easily rupture when overburdened with intracellular parasites. This can lead to a diagnostic challenge when encountering a patient with these symptoms. 

Therapeutic intervention

Therapeutic intervention for babesiosis depends on the severity of the infection. Severe babesiosis typically occurs with parasitemia levels at or above 4%, though it can also be present at levels below 4% [4]. Patients who are over the age of 50 or are immunocompromised are at higher risk for severe cases. Given the patient's age, the duration of parasitemia, severe anemia, and end-organ dysfunction, she meets the criteria for severe babesiosis. 

The preferred treatment for an immunocompetent individual with severe babesiosis involves administering intravenous azithromycin at 500 mg once daily, combined with oral atovaquone at 750 mg every 12 hours for seven to 10 days [4]. If there is a concern for co-infection with Lyme disease or anaplasmosis, doxycycline can be given at 100 mg twice daily for seven days. This is also an option for patients whose symptoms worsen or fail to improve within the first 48 hours of treatment [4].

Alternatively, treatment can include IV clindamycin at 600 mg every six hours until symptoms improve, along with oral quinine sulfate at 650 mg every eight hours. Once symptoms and parasitemia levels have decreased, intravenous therapy can transition to oral therapy. In cases of malaria, artemether/lumefantrine can be administered twice daily for three days in addition to IV therapy, or atovaquone/proguanil at 750 mg orally every 12 hours can be used. If symptoms like fever persist, therapy should be continued. Cessation of treatment should be based on the clearance of parasites and clinical improvement. Fatigue may linger even after therapy ends and should not be a reason to extend treatment [3-5].

 Initially, this patient was diagnosed with malaria and treated with quinine sulfate (two tablets three times daily) and doxycycline (100 mg IV twice daily) for one week. However, due to clinical deterioration that required intubation, both medications were paused for one day. Instead of restarting them after stabilization, she was switched to artemether/lumefantrine. On day five of her second admission, her malaria percentage was recorded at 0.56%, with the smear remaining positive. ID specialists then decided to resume doxycycline. By day eight, her malaria levels were still positive at 0.38%. At this point, ID recommended adding atovaquone/proguanil once daily for three days to her treatment regimen. 

On day 11 of the first hospitalization, the patient's malaria percentage had only decreased to 0.37%, prompting ID to send a sample for species identification to better control the infection. Once notified by the health department that the patient had babesia rather than malaria, the recommendation was made to switch her treatment to atovaquone at 750 mg twice daily and azithromycin at 500 mg once daily, both for one week. By the time the correct antibiotic regimen was initiated, the patient had already completed her courses of doxycycline and artemether/lumefantrine. Improvement was noted as her hemoglobin levels ranged from 7.7 to 8.2 over the week. However, on day seventeen, ID found that her babesia level had increased to 0.47%, compared to 0.37% just six days earlier. During a lab draw that day, her Hgb dropped to 6.9, necessitating a transfusion. The following day, after being transferred to the MedSurg unit, the patient reported feeling better than when she first arrived. Her pain was well managed. She was also noted to be feeling more lively and less fatigued.

## Discussion

This case highlights the complexity of diagnosing and managing severe babesiosis, particularly in an immunocompromised patient with multiple comorbidities. Babesiosis, a tick-borne infection caused by Babesia species, often presents with nonspecific symptoms such as fever, fatigue, and hemolytic anemia. These symptoms can mimic other conditions, leading to diagnostic and treatment delays and poorer outcomes. Our patient was initially suspected of having malaria due to intra-erythrocytic rings on peripheral blood smear, a common feature of both Plasmodium and Babesia infections. However, the persistence of symptoms alongside antimalarial treatment and the eventual species identification confirmed Babesia as the causative agent.

Severe babesiosis predominantly affects individuals over 50 years old or those with immunosuppressive conditions, as seen in this patient who has diabetes and chronic illness. Her disease course, including progressive anemia, thrombocytopenia, and end-organ dysfunction, necessitated multiple transfusions and intensive care management. The delayed diagnosis underscores the need for heightened clinical suspicion in endemic areas, pertinent travel history, or patients with persistent, unexplained hemolysis and anemia. The initial treatment with quinine and doxycycline, appropriate for malaria, was ineffective, further emphasizing the importance of high clinical suspicion, good history taking, and prompt species identification to guide targeted therapy [6-9].

This case also illustrates the challenges in managing severe babesiosis, where standard/delayed treatment with atovaquone and azithromycin may not always lead to immediate resolution. Despite appropriate therapy, our patient continued to exhibit parasitemia, requiring prolonged hospitalization and close monitoring. The need for transfusions and ICU support highlights the potential severity of babesiosis and its complications, such as acute respiratory distress syndrome and septic shock [10,11]. Moreover, the presence of concurrent autoimmune markers, including positive antinuclear antibodies (ANA) and antimitochondrial antibodies (AMA), added to the diagnostic complexity. These markers initially raised concern for autoimmune hemolytic anemia or underlying autoimmune liver disease, such as primary biliary cholangitis or systemic sclerosis. Had these autoimmune conditions been confirmed as the primary cause, management would have shifted toward immunosuppressive therapy rather than antimicrobial treatment. However, the lack of a positive Coombs test and the eventual identification of intraerythrocytic parasites pointed toward an infectious etiology, steering the clinical team away from corticosteroids or other immunosuppressive agents, which could have worsened the underlying parasitic infection.

## Conclusions

The case reinforces the importance of considering babesiosis in patients with unexplained hemolytic anemia, particularly in endemic regions or among immunocompromised individuals. The diagnostic challenge represented by its similarity to malaria emphasizes the necessity of early species differentiation through peripheral smear analysis and polymerase chain reaction testing or rapid testing. Delayed recognition and appropriate therapy can result in severe complications, including multi-organ failure and prolonged hospitalization, as seen in this patient. Clinicians should maintain a high index of suspicion for babesiosis in febrile patients with hemolysis and thrombocytopenia, especially when initial treatments fail to yield improvement or are not explained by other disease processes. Early diagnosis, appropriate antimicrobial therapy, and high clinical suspicion are key to improving outcomes in patients with severe babesiosis. This case contributes to the medical literature by revealing the diagnostic pitfalls and therapeutic challenges associated with severe babesiosis, declaring the need for timely and precise clinical decision-making.
